# Adulticidal Activity of the Insect Growth Regulators Methoprene and Cyromazine in House Flies (*Musca domestica* L.): Evidence from Feeding Trials

**DOI:** 10.3390/biology14111495

**Published:** 2025-10-26

**Authors:** Gökhan Erdoğan

**Affiliations:** Department of Organic Agriculture, Vocational School of Manavgat, Akdeniz University, 07600 Antalya, Türkiye; gokhanerdogan@akdeniz.edu.tr

**Keywords:** adulticidal effect, cyromazine, insect growth regulator, methoprene, *Musca domestica*

## Abstract

**Simple Summary:**

House flies (*Musca domestica* L.) are widespread and significant vectors of various human and animal diseases. In recent years, the development of high levels of resistance to many insecticides has made the control of house flies increasingly difficult. In this study, the acute lethal effects of insect growth regulators (IGRs), usually used to target larva stage, were evaluated by oral administration to adult house flies. The mortality rates observed within 48 h ranged from 70.62% to 100% for all concentrations and populations tested. Our results suggest that IGRs at appropriate concentrations can be effectively used in house flies control strategies and provide a basis for future research in this field.

**Abstract:**

House flies (*Musca domestica* L.) are major vectors of numerous pathogens affecting both humans and animals. The global distribution of house flies has been steadily increasing the expansion of human settlements, increased waste production, and the growth of livestock farms established to meet the demand for animal-derived products. Frequent exposure to intensive pesticide applications in agricultural and livestock areas has accelerated the development of insecticide resistance, posing a serious challenge to sustainable control efforts. The widespread and repeated use of conventional chemical insecticides has contributed to rapid resistance evolution in many populations worldwide. In this study, the acute toxic effects of two insect growth regulators (IGRs)—cyromazine and methoprene—commonly used in the larval stages of house flies were evaluated against adult flies. Treatments were applied (3 replicates) orally via 40% sugar-water solutions containing 1%, 5%, and 10% concentrations, and bioassays were conducted on eight distinct house fly populations. The results showed that cyromazine caused average adult mortalities of 76.35%, 81.00%, and 84.50% within 48 h, while methoprene produced 70.62%, 99.37%, and 100% mortality at the same concentrations. At 10%, methoprene achieved 100% mortality across all populations, whereas cyromazine induced mortality ranging from 44.28% to 100%. These findings suggest that IGRs can be effective alternatives to conventional insecticides and can be integrated into IPM/IVM programs to reduce chemical use and delay resistance.

## 1. Introduction

The global population grew from 1 billion in the early 1800s to 7 billion by 2012 and reached 8.2 billion in 2024, with projections nearing 10 billion within the next three decades [1]. The rapid growth of the human population has increased pressure on ecosystems to meet basic needs such as food and shelter, leading to intensified construction activities in urban areas and the expansion of urban settlements into rural and forested areas [2,3]. Most vector organisms are closely linked to human activity, and millions of people worldwide are affected or die each year from vector-borne diseases [4]. Adverse global factors such as climate change, population growth, uncontrolled migration, and unplanned urbanization are further intensifying the destructive impact of vectors [5]. Consequently, the control of vector insects is becoming increasingly important. Among vector insects, house flies *Musca domestica* L. (Diptera: Muscidae) hold a significant position as they are capable of transmitting a wide range of pathogens, including viruses, bacteria, and fungi, posing serious risks to both human and animal health. Cholera, dysentery, typhoid fever, and diarrheal outbreaks are among the most significant diseases caused by house flies in humans. Moreover, in areas where livestock farming is practiced, high fly population densities can induce stress in animals, leading to decreased production of meat, milk, and eggs [6,7,8].

Due to their high adaptability, wide distribution and high reproductive success, houseflies are among the insect species in which resistance development is rapidly advancing. Despite long-standing efforts to control houseflies, effective and sustainable success has not yet been achieved, and houseflies have developed resistance to various classes of insecticides, including synthetic pyrethroids, carbamates, and others [9,10,11,12,13,14,15,16,17]. Therefore, researchers and insecticide manufacturers continue to search for new strategies to overcome this resistance. One such approach involves the use of insect growth regulators (IGRs). IGRs are primarily applied during pre-adult stages, particularly as larvicides. Juvenile hormone analogues (JHAs) and chitin synthesis inhibitors (CSIs) are among the most commonly used IGR classes [16]. Chitin synthesis inhibitors (CSIs), commonly used as larvicides, disrupt molting by blocking new cuticle formation. They exert their effects through multiple mechanisms, including inhibition of chitin synthase, proteases, and UDP-N-acetylglucosamine transport [18,19]. Juvenile hormone analogs (JHAs) are most effective in early metamorphic stages, disrupting embryogenesis, causing abnormal pupation, and reducing adult emergence [20,21,22]. In addition, the oral administration of IGRs to house flies can also exhibit a chemosterilant effect by reducing both the number of eggs laid and the number of adults emerging from those eggs [23]. Cyromazine [N-cyclopropyl-1,3,5-triazine-2,4,6-triamine] is an aminotriazine IGR that affects larval and pupal cuticles in Dipterans and certain other insect species. Although the exact mode of action of cyromazine is not fully understood, it has been shown to disrupt specific steps in cuticle sclerotization [24]. Additionally, this molecule may interfere with DNA synthesis by disrupting the cellular incorporation of cytosine and adenosine [25]. Methoprene (Propan-2-yl (2E,4E)-11-methoxy-3,7,11-trimethyldodeca-2,4-dienoate), the first synthesized JHA, prevents insects from transitioning from the pupal stage to adulthood and effectively halts the biological life cycle, particularly reproduction [26]. Synthetic JHAs such as methoprene prevent metamorphosis into the adult stage when applied to immature insects, although they do not typically cause mortality in adult insects [27]. Since the 1970s, extensive laboratory research has investigated the direct toxicity of methoprene on immature insects [28,29,30,31,32]. However, more recent studies have focused on sublethal effects that better reflect actual field exposure conditions [33,34,35,36,37,38,39,40].

This study, therefore, aimed to evaluate the adulticidal efficacy of cyromazine and methoprene, two commonly used larvicidal IGRs, against resistant populations of *Musca domestica* from various Turkish provinces. In this context, the adulticidal effects of cyromazine and methoprene were tested on eight *M. domestica* populations, including seven from different provinces across five geographical regions of Türkiye and one reference population from the World Health Organization (WHO).

## 2. Materials and Methods

### 2.1. House Flies

**Figure 1:**
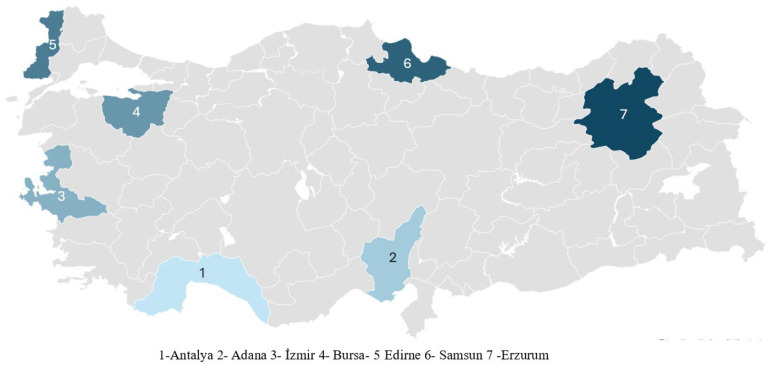
Locations where adult *M. domestica* were sampled for current study in Türkiye.

House fly (*M. domestica*) populations were collected from seven provinces—Adana, Antalya, Bursa, Edirne, Erzurum, İzmir, and Samsun—located across five geographical regions of Türkiye (Aegean, Mediterranean, Eastern Anatolia, Marmara, and Black Sea) (Figure 1, Table 1). Including the WHO laboratory population, a total of eight distinct populations were obtained. Adult house flies were captured using traps on cattle farms and transferred into mesh cages containing food and water. These specimens were then transported to the Vector Research Laboratory of the Department of Biology at Akdeniz University, Faculty of Science, where they were reared under laboratory conditions. To ensure healthy development and maintenance of the cultures, the flies were provided with an adequate diet consisting of bran, milk, and sugar at a ratio of 4:0.3:0.3:2 by weight, respectively, along with a continuous water supply [41]. All cultures were maintained under controlled environmental conditions: 26 ± 2 °C temperature, 60 ± 5% relative humidity, and a photoperiod of 12 h light and 12 h dark. Eggs deposited on cotton pads soaked in milk inside the rearing cages were carefully collected using forceps and transferred to jars covered with mesh and containing a milk-bran mixture. Upon the emergence of the first adults from these eggs, flies were transferred to new 20 × 20 × 20 cm mesh cages to continue colony maintenance.

### 2.2. Adulticidal Effect Tests

In the adulticidal effect assays, three concentrations (1%, 5%, and 10%) of cyromazine and methoprene were tested (Table 2). The test solutions were prepared by dissolving technical grade cyromazine (75% purity) and methoprene (99% purity) in a 40% (*w*/*v*) sucrose solution to obtain final active ingredient concentrations of 1%, 5%, and 10% (*w*/*v*). For 1 L of each solution, 13.33, 66.67, and 133.33 g of cyromazine, and 10.10, 50.51, and 101.01 g of methoprene were weighed and dissolved, respectively. The prepared solutions were absorbed onto cotton pads, which were then placed into glass jars (1 L capacity) containing adult *M. domestica* individuals. New 1 L glass jars that had never been used before were used in each trial. The solutions were prepared fresh before each biological experiment to prevent microbial growth or crystallization during storage. In all experiments, 2–4-day-old mixed-sex F_1_ adults obtained from field-collected populations were used. Cotton pads soaked in solutions containing 1%, 5%, and 10% concentrations dissolved in a 40% sugar-water solution were placed into jars with a volume of 1 L, and at least 25 adult house flies were released into each jar. Mortality was recorded at 24 and 48 h post-exposure. In the control group, only cotton pads soaked in sugar solution without any IGRs were used, and 25 adults were placed into each jar. All treatments, including the control, were conducted in three replicates. All bioassays were conducted under controlled laboratory conditions maintained at 26 ± 2 °C, with a relative humidity of 60 ± 5%, and a 12:12 h light/dark photoperiod [42].

### 2.3. Statistical Analysis

Mortality percentages were subjected to arcsine square-root transformation prior to statistical analysis to stabilize variances and approximate normality [44]. The assumptions of normality and homogeneity of variances were verified using the Shapiro–Wilk and Levene’s tests, respectively. The transformed data met ANOVA assumptions. The transformed data were analyzed using one-way analysis of variance (ANOVA) in SPSS software version 23.0 (IBM Corp., Armonk, NY, USA). When significant differences were detected among treatments (*p* ≤ 0.05), means were separated using Tukey’s HSD (Honestly Significant Difference) post hoc test. The results are presented as mean ± standard error (SE), and means followed by the same letter were not significantly different at the 5% probability level [13,23].

## 3. Results

As a result, the 1% concentration of cyromazine caused an 36.45% mortality after 24 h, with the lowest mortality observed in the İzmir population (3.72%) and the highest in the Adana population (65.55%). At 48 h, the mortality rate increased to 76.35%, ranging from 38.95% in the İzmir population to 100% in the WHO reference population. At the 5% concentration of cyromazine, the mortality rate at 24 h was 46.89%, ranging from 9.17% (İzmir) to 85.81% (WHO). At 48 h, the mortality rate reached 81.00%, with the lowest value recorded in the Bursa population (45.41%) and the highest again in the WHO population (100%). For the 10% concentration, the 24 h average mortality rate was 54.45%, varying from 10.55% in the Bursa population to 89.23% in the WHO population. After 48 h, the average mortality rate increased to 84.495%, with a minimum of 44.28% (Bursa) and a maximum of 100% observed in the Erzurum and WHO populations (Table 3).

Regarding the results of methoprene, the 1% concentration produced an average mortality rate of 58.26% at 24 h, with the lowest mortality observed in the Antalya population (10.81%) and the highest in the Erzurum population (97.06%). At 48 h, the mortality rate increased to 70.62%, ranging from 34.69% (Antalya) to 100% in the WHO population. At the 5% concentration, methoprene caused an average mortality of 96.84% at 24 h, ranging from 86.35% (Antalya) to 100% (Edirne). After 48 h, the mortality rate reached 99.37%, ranging from 98.55% (Erzurum) to 100% in the WHO and Adana populations. For the 10% concentration, the average mortality at 24 h was 99.56%, with 96.47% mortality in the Antalya population and 100% in the remaining seven populations. At 48 h, a 100% mortality rate was recorded across all tested populations (Table 4).

When the data were evaluated in terms of populations, it was found that for cyromazine at 24 h, the Bursa population exhibited no statistically significant difference in mortality rates compared to the control group at any of the three tested concentrations. The Bursa population was followed by the İzmir and Erzurum populations, which also showed the lowest mortality rates. The highest mortality rates were recorded as follows: 65.55% in Adana at the 1% concentration, 85.81% in the WHO population at the 5% concentration, and 100% in both the Erzurum and WHO populations at the 10% concentration. At 48 h, cyromazine caused statistically significant mortality in all provinces and at all concentrations when compared to the control. The lowest mortality rates were observed in İzmir (38.95%) at the 1% concentration, Bursa (45.41%) at the 5% concentration, and Bursa (44.28%) again at the 10% concentration. The highest mortality rates at 48 h were found in the WHO population, with 100% mortality observed at all three concentrations (Table 4).

**Figure 2:**
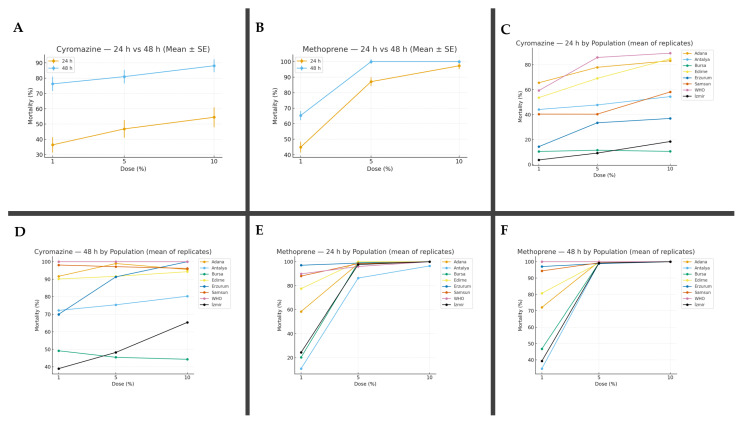
Dose–response relationships and mortality variation among Musca domestica populations treated with insect growth regulators (IGRs). Mean adult mortality (Mean ± SE) of eight M. domestica populations exposed to increasing concentrations (1%, 5%, 10%) of Cyromazine (**A**) and Methoprene (**B**) after 24 h and 48 h of exposure. Mortality responses of individual populations to Cyromazine (**C**,**D**) and Methoprene (**E**,**F**) at 24 h and 48 h, respectively.

For methoprene, statistically significant differences in mortality rates compared to the control group were observed at all concentrations and all exposure times. According to the 24 h results, the Antalya population exhibited the lowest mortality rates across all concentrations: 10.81% at 1%, 86.35% at 5%, and 96.47% at 10%. The highest mortality rates were recorded as 97.06% in Erzurum at the 1% concentration, 100% in Edirne at the 5% concentration, and 100% in all populations except Antalya at the 10% concentration. In terms of the 48 h results, the lowest mortality rate was observed in Antalya at the 1% concentration (34.69%) and in Erzurum at the 5% concentration (98.85%). However, no statistically significant difference was found between the mortality rate of Erzurum and those of the Antalya, Samsun, İzmir, Edirne, and Bursa populations at the same concentration level. At the 10% concentration, a 100% mortality rate was observed in all tested populations. Overall, the Antalya and Bursa populations, particularly at the 1% concentration, stood out as the populations with the lowest mortality rates for methoprene (Table 5, Figure 2).

## 4. Discussion

Currently, large-scale human and animal migrations are occurring due to global climate change, as well as political and socio-economic factors. With global climate change, the distribution ranges of vectors are expanding, and it is known that some species are beginning to migrate from warming regions to more temperate zones [45,46]. In parallel, major human migrations have contributed to the increased spread of many zoonotic and vector-borne diseases [47,48]. Türkiye, is located at the crossroads of three continents, is highly likely to serve as a convergence point for the movement of humans, animals, and vector insects [49]. For this reason, the importance of vector control is increasing. However, the development of resistance to conventional control methods has led to serious challenges in vector management. To overcome these issues, it is essential to promote the integrated use of multiple control strategies rather than relying on a single method.

Today, the use of certain insecticide groups such as organochlorines and organophosphates has been completely banned in both public health and agricultural sectors in Türkiye due to their negative impacts on non-target organisms and the environment. Among the insecticides that are still in use, increasing environmental concerns and the emergence of resistance have become significant issues [50,51,52,53,54,55,56,57,58,59,60,61,62,63,64,65,66,67,68,69]. As a consequence, there is a growing risk of greater agricultural yield losses and the occurrence of severe vector-borne diseases in Türkiye. In response, researchers are actively investigating alternative insecticides and novel methods that are effective against pest species [57]. Among these alternatives, insect growth regulators (IGRs) have gained prominence. The most widely used IGRs are JHAs and CSIs. Natural juvenile hormone (JH) regulates critical processes such as development, reproduction, diapause, and polyphenism throughout an insect’s life and exerts its effects on nearly all tissues. Therefore, fluctuations in JH titers at specific stages of the life cycle can disrupt normal metamorphosis. Induction of JH levels can also trigger a cascade of disruptions in other hormonally regulated physiological functions. CSIs, which are mainly used as larvicides in vector control, inhibit the polymerization of N-acetylglucosamine, a process catalyzed by the chitin synthase enzyme. The application of CSIs typically results in mortality or morphological abnormalities during the larval and pupal stages, and in some cases, at adult emergence. The duration of exposure and the developmental stage of the insect significantly influence the efficacy of CSIs. When exposure occurs in the late larval stage, adult emergence may still occur; however, deformities in body structures such as wings and legs can be observed. Moreover, studies have demonstrated that oral administration of IGRs to adult insects can exert chemosterilant effects by disrupting reproduction [13,23,58,59,60,61].

In the literature reviewed, no studies were found that investigated the adulticidal effects of orally administered methoprene and cyromazine on adult house flies. Therefore, this study represents the first investigation in this specific area. Studies conducted on other insect species have generally shown that IGRs exhibit low adulticidal effects at low concentrations, while mortality rates tend to increase as the applied concentration increases [62,63,64,65,66,67,68,69].

There are limited studies available on the lethal effects of IGRs on adult insects. In a study conducted by Ebeid et al. [62], the acute toxicity and effects of Cyfluthrin, Profenofos, and Runner (Methoxyfenozide, 240 SC) on the activity of *Bracon hebetor* S. (Hymenoptera: Braconidae) were investigated. The results revealed that Runner had an LC_50_ value of 10.874 µL/80 mL water and an LC_90_ value of 8958.338 µL/80 mL water in adult individuals. These findings support our results in that mortality rates increased with increasing concentrations, and a mortality rate approaching 90% was achieved at the 10% concentration level. In another study, the lethal and sublethal effects of three IGRs—novaluron, methoxyfenozide, and pyriproxyfen—were evaluated on *Palpita unionalis* H. (Lepidoptera: Pyralidae), as well as the demographic toxicology of these IGRs on its parasitoid *H. hebetor*. The LC_50_ values of these IGRs for *P. unionalis* were found to be 0.97, 0.176, and 0.00009 ppm, respectively, indicating that pyriproxyfen was the most toxic [63]. After determining effective doses in that study, the adulticidal potential of the IGRs was observed, which also supports our findings. The fact that IGRs showed adulticidal effects at lower doses in *P. unionalis* compared to *M. domestica* may be attributed to the more limited use of IGRs in agricultural settings. While house flies are exposed to a broader range of insecticides due to their widespread presence in agricultural, livestock, and urban environments, agricultural pests such as *P. unionalis* are generally confined to specific areas and thus encounter a more limited spectrum of chemical exposure. In another study conducted by Sampson et al. [64], combinations and individual applications of 1 ppm and 0.5 ppm lufenuron and 100 ppm erythritol were tested on *Drosophila suzukii* M. (Diptera: Drosophilidae) adults using four small blackberry fruits as the substrate. In the trials where lufenuron was applied alone, mortality rates reached 48% in males and 57% in females. When lufenuron was combined with erythritol, mortality increased to 82.2% in males and 78.3% in females. The reason for mortality rates close to 50% at relatively low doses is thought to be due to the difference in the insect species used in the experiment.

The adulticidal effects of pyriproxyfen (1 mg/L) in combination with either 1% boric acid sugar bait or 5% eugenol sugar bait were evaluated against *Aedes albopictus* S. (Diptera: Culicidae) by spraying the mixtures onto Croton petra plants (*Codiaeum variegatum* L.). In the trials, *A. albopictus* adults that came into contact with plants treated with pyriproxyfen and 1% boric acid sugar bait exhibited a 63.3% mortality rate, while those exposed to plants treated with pyriproxyfen and 5% eugenol sugar bait exhibited 80.4% mortality [65]. These findings support the results of this study.

In a study evaluating the combined application of diatomaceous earth (DE) (Protect-It^®^) and methoprene (Diacon^®^ II) against the lesser grain borer *Rhyzopertha dominica* F. (Coleoptera: Bostrichidae) in stored rough rice, mortality rates for the combination of 1 ppm methoprene and 500 ppm DE were reported as 77.5 ± 9.0% in long-grain rice, 77.5 ± 10.0% in medium-grain rice, and 58.5 ± 3.0% in short-grain rice. In contrast, in treatments where methoprene was not included, mortality rates were significantly lower, with 57.5 ± 12.0% and 58.8 ± 9.7% for long- and medium-grain rice, respectively, and only 26.3 ± 4.7% for short-grain rice [66]. These findings support the adulticidal efficacy of methoprene and are in agreement with the results of this study.

In a study conducted by Nisar et al. [67], adult *Bactrocera zonata* S. (Diptera: Tephritidae) individuals were orally exposed to various IGRs, including methoxyfenozide, fenoxycarb, lufenuron, pyriproxyfen, and buprofezin. The resulting mortality rates were reported as 37.5% (0.44% LC_50_), 34.7% (0.50% LC_50_), 30.9% (0.50% LC_50_), 25.1% (0.97% LC_50_), and 22.5% (1.05% LC_50_), respectively in this study as well, the lower mortality rates and effective doses observed compared to our findings are likely due to differences in insect species and the relatively lower likelihood of prior insecticide exposure compared to house flies.

In a study conducted by Mahmoud et al. [68], adult *Rhyzopertha dominica* F. (Coleoptera: Bostrichidae) were provided with wheat grains treated with different concentrations (0.2, 0.4, and 0.8 ppm) of buprofezin, hexaflumuron, and lufenuron. The results demonstrated that adult mortality increased with both concentration and exposure duration. After 21 days of treatment, the highest mortality rates (80.00%, 78.33%, and 60.00%) were recorded at 0.8 ppm, whereas the lowest mortality rates (58.33%, 46.66%, and 30.00%) were observed at 0.2 ppm for lufenuron, buprofezin, and hexaflumuron, respectively. In the study conducted by Ali et al. [69] on the red flour beetle, *Tribolium castaneum* H. (Coleoptera: Tenebrionidae), serial concentrations of insect growth regulators were tested, ranging from 2.5 to 20 ppm. The results demonstrated that at the highest tested concentration (20 ppm), lufenuron caused 33.3% mortality, while methoxyfenozide induced 23.3% mortality. In contrast, pyriproxyfen did not result in any adult mortality at any of the tested concentrations. These findings indicate that, although some IGRs can exert adulticidal effects in *T. castaneum*, their overall efficacy is limited and strongly dependent on both the compound and concentration applied. These interspecific differences may be attributed to the modes of action and metabolic capacities of each species. Methoprene, a juvenile hormone (JH) analogue, disrupts hormonal balance in adults, inducing rapid metabolic and physiological stress that leads to high mortality in a short time. Cyromazine, a chitin synthesis inhibitor, can also affect adult stages through metabolic and structural disruptions, rather than exclusively targeting larvae. The lower mortality rates observed in Mahmoud’s and Ali’s studies are likely influenced by the specific IGR used, dosage, exposure duration, and the species’ detoxification capabilities. These studies collectively demonstrate that IGRs can exert adulticidal effects at varying concentrations and exposure durations depending on the insect species, and they support the findings of this study. In these experiments, that the inclusion of 5–10% cyromazine and methoprene in sugar-based bait formulations resulted in effective control of adult house flies. At a time when insecticide resistance is significantly increasing, this approach demonstrates a promising alternative for house flies’ management. Moreover, it is anticipated that incorporating these IGRs into bait formulations enriched with sugar water or attractants containing stronger aromas could enhance attractiveness and broaden the spectrum of target species. As a result, these baits could provide adulticidal or chemosterilant effects and may help reduce insect densities—particularly in high-infestation environments such as barns and animal shelters. However, it should be noted that prolonged or repeated exposure to IGR-based baits could potentially promote resistance development in field populations. Therefore, future studies should incorporate field validation trials and long-term resistance monitoring to confirm laboratory findings and ensure the sustainable use of IGRs in integrated pest management programs.

## 5. Conclusions

In this study, IGRs, which are commonly used as larvicides, were administered orally to adult house flies to evaluate their adulticidal effects. The experiments were conducted on a total of eight populations, including seven field populations and one laboratory population. The results indicated that both cyromazine and methoprene exhibited high acute adulticidal effects at 5% and 10% concentrations within 48 h. In an era marked by widespread insecticide resistance, this method is considered a promising alternative for housefly control. A comprehensive review of the literature revealed no prior studies evaluating the adulticidal effects of IGRs on house flies via oral administration, to my knowledge. Implementing this approach as an ingestible bait rather than via surface spraying may significantly reduce the risk of non-target exposure and associated adverse effects on humans, domestic animals, and other non-target organisms in house flies management. This study holds significance as the first to explore this approach, and it has the potential to serve as a valuable reference for future research in this field. On the other hand, a limitation of this study is that the laboratory setup does not fully represent field conditions. Environmental factors such as temperature fluctuations, sunlight exposure, and substrate interactions that may influence the degradation or persistence of insect growth regulators (IGRs) were not considered. Additionally, variations in feeding behavior and bait contact under natural settings could alter mortality outcomes compared to controlled laboratory assays. Another limiting factor of this study is that adult house flies were used without sex differentiation. Mixed-sex individuals were tested randomly, and the possible influence of sex on feeding behavior or insecticide susceptibility was not specifically evaluated. Although sex-related differences have been reported in some insect species, mixed-sex cohorts are commonly used in operational bioassays, and this approach is considered acceptable for general adulticidal evaluation. Therefore, further field evaluations are necessary to validate the laboratory findings and assess the real-world efficacy of methoprene and cyromazine treatments.

## Figures and Tables

**Table 1:** Locations of house fly collections from the field.

Locality	District	Coordinates	Sampling Date
Adana	Ceyhan	36.90413° N–35.94073° E	June 2021
Antalya	Kepez	36.99272° N–30.71738° E	June 2021
Bursa	Nilüfer	40.16571° N–28.72822° E	July 2021
Edirne	Merkez	41.62572° N–26.56585° E	July 2021
Erzurum	Aşkale	39.85275° N–40.60816° E	August 2021
İzmir	Çeşme	38.31391° N–26.47018° E	July 2021
Samsun	İlkadım	41.28796° N–36.20145° E	August 2021

**Table 2:** Information on insect growth regulators used in the study [43].

Name	Mode of Action	Cas Number	IUPAC Name
Cyromazine	CSI	66215-27-8	2-*N*-cyclopropyl-1,3,5-triazine-2,4,6-triamine
Methoprene	JHA	40596-69-8	propan-2-yl (2*E*,4*E*)-11-methoxy-3,7,11-trimethyldodeca-2,4-dienoate

**Table 3:** Adulticidal effects of cyromazine (cyro) (%mortality ± standard error).

IGR	Antalya		Erzurum		Samsun		İzmir		Edirne		Bursa		Adana		WHO	
	24 h	48 h	24 h	48 h	24 h	48 h	24 h	48 h	24 h	48 h	24 h	48 h	24 h	48 h	24 h	48 h
Cyro %1	44.14 ± 7.16 B,b	72.16 ± 7.54 C,b	14.37 ± 0.83 B,b	69.91 ± 4.80 C,b	40.42 ± 7.48 B,b	98.13 ± 1.80 C,b	3.72 ± 0.79 A,a	38.95 ± 4.08 B,b	53.64 ± 3.06 B,b	90.80 ± 2.28 C,b	10.48 ± 1.54 A,b	49.15 ± 8.85 B,b	65.55 ± 4.93 B,b	91.67 ± 4.74 C,b	59.31 ± 21.33 B,b	100 ± 0.00 C,b
Cyro %5	47.76 ± 1.51 B,b	75.35 ± 5.30 C,b	33.45 ± 3.49 B,c	91.33 ± 4.67 C,bc	40.38 ± 5.68 B,b	97.16 ± 1.58 C,b	9.17 ± 2.88 B,b	48.20 ± 1.55 C,bc	69.04 ± 3.33 B,c	91.61 ± 2.27 C,b	11.49 ± 4.99 B,b	45.41 ± 2.29 C,b	77.93 ± 3.62 B,bc	98.96 ± 1.66 C,b	85.81 ± 5.91 B,bc	100 ± 0.00 C,b
Cyro%10	54.46 ± 1.69 B,b	80.26 ± 2.15 B,b	36.93 ± 8.41 B,c	100 ± 0.00 C,c	58.17± 19.80 B,b	96.16 ± 1.00 B,b	18.46 ± 1.54 A,c	65.38 ± 9.88 B,c	84.65 ± 2.48 B,d	94.28 ± 0.36 C,b	10.55 ± 5.96 A,b	44.28 ± 9.04 B,b	83.17 ± 6.39 B,c	95.56 ± 4.81 C,b,	89.23 ± 6.31 B,c	100 ± 0.00 C,b
Control	0.00 ± 0.00 A,a,	0.00 ± 0.00 A,a,	0.00 ± 0.00 A,a,	0.00 ± 0.00 A,a,	0.00 ± 0.00 A,a,	0.00 ± 0.00 A,a,	0.00 ± 0.00 A,a,	0.00 ± 0.00 A,a,	0.00 ± 0.00 A,a,	0.00 ± 0.00 A,a,	0.00 ± 0.00 A,a,	0.00 ± 0.00 A,a,	0.00 ± 0.00 A,a,	0.00 ± 0.00 A,a,	0.00 ± 0.00 A,a,	0.00 ± 0.00 A,a,

If the uppercase letters in the same column differ, it indicates a statistically significant difference between time points for the same concentration within each province *p* ≤ 0.05. If the lowercase letters in the same row differ, it indicates a statistically significant difference between different concentrations of the same active ingredient *p* ≤ 0.05.

**Table 4:** Adulticidal effects of methoprene (metho) (%mortality ± standard error).

IGR	Antalya		Erzurum		Samsun		İzmir		Edirne		Bursa		Adana		WHO	
	24 h	48 h	24 h	48 h	24 h	48 h	24 h	48 h	24 h	48 h	24 h	48 h	24 h	48 h	24 h	48 h
Metho%1	10.81± 1.38 A.b	34.69 ± 12.6 B,b	97.06± 1.65 B,b	97.06 ± 1.65 B,b	88.01 ± 1.92 B,b	94.37 ± 3.40 B,b	24.30 ± 4.72 B,b	39.33 ± 7.47 B,b	77.49 ± 6.59 B,b	80.69 ± 6.66 B,b	20.20 ± 8.41 A,b	46.76 ± 8.48 B,b	58.37 ± 22.60 B,b	72.06 ± 5.80 B,b	89.86 ± 5.22 B,b	100 ± 0.00 C,b
Metho %5	86.35 ± 2.28 B,c	99.15± 0.83 C,c	98.85 ± 1.11 B,b	98.85 ± 1.11 B,b	98.37 ± 1.57 B,c	99.32 ± 0.70 B,b	97.65 ± 1.42 B,c	99.19 ± 0.82 C,c	100 ± 0.00 B,c	99.26 ± 0.72 B,c	99.17 ± 0.83 B,c	99.17 ± 0.83 B,c	98.33 ± 2.50 B,c	100 ± 0.00 B,c	96.00 ± 5.77 B,c	100 ± 0.00 B,b
Metho%10	96,47 ± 0.63 B,d	100 ± 0.00 C,c	100 ± 0.00 B,b	100 ± 0.00 B,b	100 ± 0.00 B,c	100 ± 0.00 B,b	100 ± 0.00 B,c	100 ± 0.00 B,c	100 ± 0.00 B,c	100 ± 0.00 B,c	100 ± 0.00 B,c	100 ± 0.00 B,c	100 ± 0.00 B,c	100 ± 0.00 B,c	100 ± 0.00 B,c	100 ± 0.00 B,b
Control	0.00 ± 0.00 A,a,	0.00 ± 0.00 A,a,	0.00 ± 0.00 A,a,	0.00 ± 0.00 A,a,	0.00 ± 0.00 A,a,	0.00 ± 0.00 A,a,	0.00 ± 0.00 A,a,	0.00 ± 0.00 A,a,	0.00 ± 0.00 A,a,	0.00 ± 0.00 A,a,	0.00 ± 0.00 A,a,	0.00 ± 0.00 A,a,	0.00 ± 0.00 A,a,	0.00 ± 0.00 A,a,	0.00 ± 0.00 A,a,	0.00 ± 0.00 A,a,

If the uppercase letters in the same column differ, it indicates a statistically significant difference between time points for the same concentration within each province *p* ≤ 0.05. If the lowercase letters in the same row differ, it indicates a statistically significant difference between different concentrations of the same active ingredient *p* ≤ 0.05.

**Table 5:** Adulticidal effects of cyromazine and methoprene on various house fliy populations in Türkiye (%mortality ± standard error).

Populations	Cyromazine %1		Cyromazine %5		Cyromazine %10		Methoprene %1		Methoprene %5		Methoprene %10	
	24 h	48 h	24 h	48 h	24 h	48 h	24 h	48 h	24 h	48 h	24 h	48 h
Control	0.00 ± 0.00 A	0.00 ± 0.00 A	0.00 ± 0.00 A	0.00 ± 0.00 A	0.00 ± 0.00 A	0.00 ± 0.00 A	0.00 ± 0.00 A	0.00 ± 0.00 A	0.00 ± 0.00 A	0.00 ± 0.00 A	0.00 ± 0.00 A	0.00 ± 0.00 A
Antalya	44.14 ± 7.16 CD	72.16 ± 7.54 CD	47.76 ± 1.51 CD	75.35 ± 5.30 CD	54.46 ± 1.69 BCDE	80.26 ± 2.15 CD	10.81± 1.38 AB	34.69 ± 12.06 B	86.35 ± 2.28 B	99.15 ± 0.83 B	96.47 ± 0.63 B	100 ± 0.00 B
Erzurum	14.37 ± 0.83 BC	69.91 ± 4.80 CD	33.45 ± 3.49 C	91.33 ± 4.67 EF	36.93 ± 8.41 BCD	100 ± 0.00 E	97.06± 1.65 E	97.06 ± 1.65 DE	98.85 ± 1.11 BC	98.85 ± 1.11 B	100 ± 0.00 C	100 ± 0.00 B
Samsun	40.42 ± 7.48 CD	98.13 ± 1.80 EF	40.38 ± 5.68 C	97.16 ± 1.58 E	58.17± 19.80 CDE	96.16 ± 1.00 DE	88.01 ± 1.92 DE	94.37 ± 3.40 DE	98.37 ± 1.57 BC	99.32 ± 0.70 B	100 ± 0.00 C	100 ± 0.00 B
İzmir	3.72 ± 0.79 AB	38.95 ± 4.08 B	9.17 ± 2.88 B	48.20 ± 1.55 BC	18.46 ± 1.54 ABC	65.38 ± 9.88 BC	24.30 ± 4.72 BC	39.33 ± 7.47 BC	97.65 ± 1.42 BC	99.19 ± 0.82 B	100 ± 0.00 C	100 ± 0.00 B
Edirne	53.64 ± 3.06 D	90.80 ± 2.28 DE	69.04 ± 3.33 DE	91.61 ± 2.27 EF	84.65 ± 2.48 DE	94.28 ± 0.36 DE	77.49 ± 6.59 DE	80.69 ± 6.66 CDE	100 ± 0.00 C	99.26 ± 0.72 B	100 ± 0.00 C	100 ± 0.00 B
Bursa	10.48 ± 1.54 AB	49.15 ± 8.85 BC	11.49 ± 4.99 B	45.41 ± 2.29 B	10.55 ± 5.96 AB	44.28 ± 9.04 B	20.20 ±8.41 B	46.76 ± 8.48 BC	99.17 ± 0.83 C	99.17 ± 0.83 B	100 ± 0.00 C	100 ± 0.00 B
Adana	65.55 ± 4.93 D	91.67 ± 4.74 DEF	77.93 ± 3.62 E	98,96 ± 1.66 E	83.17 ± 6.39 DE	95.56 ± 4.81 E	58.37 ± 22.60 CD	72.06 ± 5.80 BCD	98.33 ± 2.50 BC	100 ± 0.00 B	100 ± 0.00 C	100 ± 0.00 B
WHO	59.31 ± 21.33 D	100 ± 0.00 E	85.81 ± 5.91 E	100 ± 0.00 E	89.23 ± 6.31 E	100 ± 0.00 E	89.86 ± 5.22 DE	100 ± 0.00 E	96.00 ± 5.77 BC	100 ± 0.00 B	100 ± 0.00 C	100 ± 0.00 B
	F: 23.161 *p*: <0.001	F: 67.309 *p*: <0.001	F: 67.024 *p*: <0.001	F: 74.776 *p*: <0.001	F: 67.024 *p*: <0.001	F: 13.759 *p*: <0.001	F: 77.852 *p*: <0.001	F: 31.586 *p*: <0.001	F: 25.179 *p*: <0.001	F: 62.386 *p*: <0.001	F: 6730.51 *p*: <0.001	F: - *p*: <0.001

If the uppercase letters in the same column differ, it indicates a statistically significant difference between provinces *p* ≤ 0.05.

## Data Availability

Data is contained within this article.
